# Discovery of *Staphylococcus aureus* small RNAs highly expressed during human chronic infection

**DOI:** 10.1128/mbio.00637-26

**Published:** 2026-08-07

**Authors:** Paul Briaud, Julia Schumacher, Marvin Whiteley

**Affiliations:** 1 School of Biological Sciences, Georgia Institute of Technology 123387, Atlanta, Georgia, USA; 2 Center for Microbial Dynamics and Infection, Georgia Institute of Technology 1372, Atlanta, Georgia, USA; 3 Emory-Children’s Cystic Fibrosis Center, Atlanta, Georgia, USA; The University of North Carolina at Chapel Hill, Chapel Hill, North Carolina, USA

**Keywords:** *Staphylococcus aureus*, chronic infection, small regulatory RNA (sRNA), zinc, metatranscriptomic, pangenome, *in vivo* gene expression, chronic wound infection, cystic fibrosis, zinc homeostasis

## Abstract

Understanding bacterial gene expression in natural environments remains challenging because most regulatory networks are inferred from *in vitro* models that poorly capture *in situ* environmental conditions. Here, we present NEMO (network transcriptomics of microbes in native environments), a generalizable pangenome-based metatranscriptomic framework for extracting microbial transcriptional programs from metatranscriptomes generated from natural samples. We applied NEMO to *Staphylococcus aureus* metatranscriptomes from human chronic wounds and cystic fibrosis sputum, revealing infection-associated transcriptional states that diverged markedly from *in vitro* growth conditions. Notably, small RNAs (sRNAs) were key drivers of the transcriptional divergence between human infection-derived and *in vitro* transcriptomes. Among these, we identify *rsaX20* as a previously uncharacterized, zinc-responsive sRNA that also encodes a small peptide. Meta-analysis of more than 2,000 publicly available *S. aureus* RNA-seq data sets showed that *rsaX20* is induced under zinc limitation and host-associated stress and is co-regulated with known metal-responsive sRNAs. Genetic, transcriptomic, and proteomic analyses demonstrated that *rsaX20* is repressed by the zinc regulator Zur and functions as a dual-purpose sRNA/small open reading frame, with the RNA and peptide exerting distinct, sometimes opposing, effects on target proteins. Together, these findings establish NEMO as a broadly applicable framework for interrogating microbial gene regulation directly from native metatranscriptomes and identify *rsaX20* as a key regulator of zinc homeostasis during chronic human *S. aureus* infection.

**IMPORTANCE:**

Most knowledge of bacterial gene regulation comes from laboratory cultures, yet pathogens behave very differently inside the human body. We introduce NEMO, a pangenome-based framework that enables direct analysis of microbial gene expression from natural infection samples. Applying NEMO to human *Staphylococcus aureus* infections revealed transcriptional programs that differ substantially from standard *in vitro* models and highlighted small regulatory RNAs as major drivers of this divergence. Using this approach, we discovered *rsaX20*, a previously uncharacterized zinc-responsive regulatory RNA that also encodes a small peptide with distinct biological functions. These findings demonstrate the power of studying microbes in their native environments and uncover a key regulator of zinc homeostasis during chronic human infection.

## INTRODUCTION

Pathogenic bacteria rely on complex regulatory networks to control protein expression during infection. A central component of this control is transcriptional regulation, in which transcription factors coordinate gene expression programs in response to environmental cues encountered within the host. These cues include fluctuations in nutrient availability, oxygen tension, pH, immune pressures, and metal levels (1, 2). Transition metals such as iron, zinc, and manganese serve as critical cues, with metal-sensing transcriptional regulators directly detecting intracellular metal availability, often through cofactor binding. During infection, the host actively restricts the availability of these transition metals through a process termed nutritional immunity, in which immune effectors such as calprotectin sequester zinc and manganese at sites of infection to limit bacterial proliferation (3, 4). As a result, *Staphylococcus aureus* must sense and respond to host-imposed metal restriction to establish and maintain infection, making metal-responsive gene regulation a central component of staphylococcal pathogenesis.

To complement transcriptional control, bacteria also employ post-transcriptional regulatory mechanisms. Small regulatory RNAs (sRNAs) constitute a major layer of post-transcriptional control. In many cases, sRNAs typically act through base pairing with target mRNAs to alter transcript stability or block translation by occluding ribosome-binding sites. Because sRNAs act directly on existing transcripts and often have short half-lives, they enable rapid modulation of protein synthesis (5). In contrast to transcriptional regulation, which often functions primarily in an on/off manner, sRNAs can fine-tune protein output by adjusting translational efficiency or mRNA stability without impacting transcript production. Well-characterized examples include the *Escherichia coli* sRNA *SgrS*, which rapidly represses *ptsG* translation (6), and the *Staphylococcus aureus* sRNA *IsrR*, which limits the synthesis of iron-containing proteins during iron limitation (7, 8). Thus, sRNAs provide a dynamic regulatory layer that allows bacteria to calibrate protein expression in fluctuating host environments.

Despite their regulatory potential, relatively few sRNAs have been functionally characterized. In *S. aureus*, hundreds of sRNAs have been predicted, with estimates ranging from 300 to over 500 depending on annotation criteria, yet only a small subset have known functions (9). This contrasts with *Escherichia coli*, which, despite having a larger genome, encodes approximately 100–150 sRNAs (10, 11), suggesting that sRNA-mediated regulation may be a more substantial player in regulating protein production in *S. aureus*. Notably, only 13 *S. aureus* sRNAs have been experimentally characterized, representing approximately 2%–4% of the predicted repertoire (7, 8, 12–23). Most studies of *S. aureus* sRNAs rely on *in vitro* experiments in which bacteria are grown under artificial conditions, such as antibiotic exposure, oxidative stress, temperature shifts, or nutrient and metal limitation in rich or defined media. While these approaches have successfully identified sRNAs and their functions, they are limited in their ability to recapitulate the complex and dynamic environments encountered during human infection (24).

Consequently, the *in vivo* expression and functional relevance of most sRNAs during human infection remain largely unknown. This gap arises because conventional workflows do not establish whether a given sRNA is expressed or functionally important during infection in humans. To address this limitation, we developed NEMO (network transcriptomics of microbes in native environments), a metatranscriptomic pipeline that enables characterization of microbial gene expression directly from human infection samples without prior bacterial isolation or culture and enables direct comparison of *in vivo* and *in vitro* gene expression (25–27). We used NEMO to define the sRNA expression landscape of *S. aureus* during chronic human wound infection and cystic fibrosis (CF) lung infection. Using a curated *S. aureus* pangenome and decoy-based filtering, NEMO revealed infection-specific transcriptional signatures that diverge markedly from *in vitro* growth conditions and demonstrated that sRNAs account for a substantial proportion of the transcriptional divergence between human infection-derived and *in vitro* transcriptomes. Nine bona fide sRNAs were consistently induced in human samples, including *rsaX20*, a previously uncharacterized sRNA that also encodes a small peptide. Integrated meta-analysis of public RNA-seq data sets, together with genetic, transcriptomic, and proteomic analyses, revealed that *rsaX20* is repressed by the zinc uptake regulator Zur and functions as a dual-purpose sRNA and small open reading frame (sORF) that coordinately regulates metal-binding protein production under zinc limitation. Together, these findings define a zinc-sparing adaptive program associated with *rsaX20* and establish pangenome-based metatranscriptomics as a powerful approach for identifying infection-relevant sRNAs highly expressed during human disease.

## RESULTS

### The NEMO workflow

**Fig 1:**
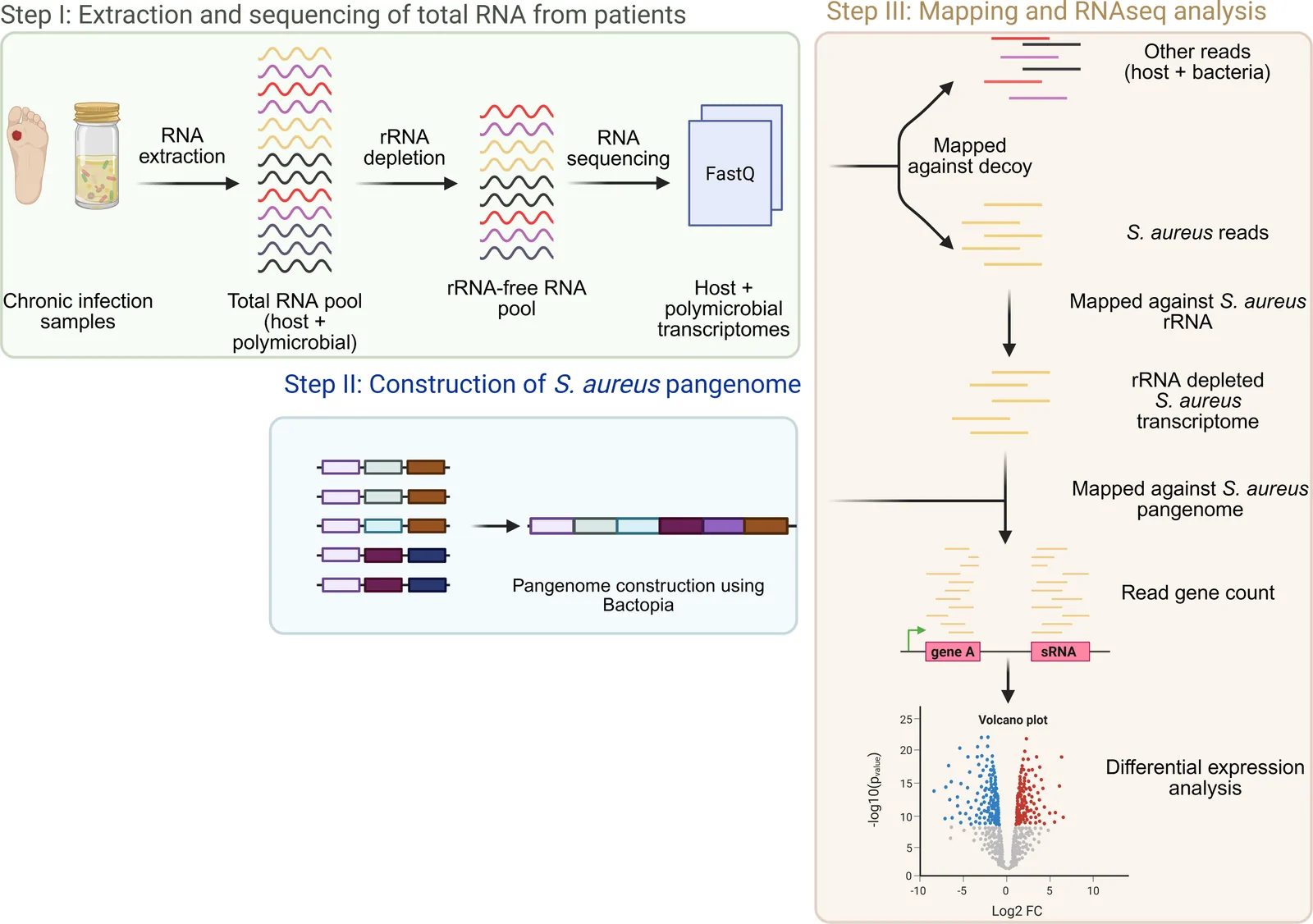
Diagram of the NEMO (network transcriptomics of microbes in native environments) framework. The NEMO approach comprises three steps. (I) Extraction and sequencing of total RNA from patient-derived infection samples. Rapid preservation of samples in an RNA-stabilizing agent (e.g., RNAlater) is essential prior to RNA extraction using mechanical (bead beating), enzymatic (lysozyme, lysostaphin), and chemical (RNA STAT-60) methods. (II) Construction of the *S. aureus* pangenome. (III) Establishing read length thresholds and accurate mapping to the *S. aureus* pangenome in polymicrobial human infection transcriptomes. Following RNA sequencing, reads are sequentially mapped to (i) a decoy database composed of genomes from the 110 most frequently isolated bacteria from human chronic wounds and CF sputum; (ii) *S. aureus* ribosomal RNA (rRNA) genes to remove rRNA reads; and (iii) the *S. aureus* pangenome constructed during step II. Principal component analysis (PCA) and differential expression analysis are then performed by comparing *in vivo* gene expression with control cultures grown *in vitro*.

**Fig 2:**
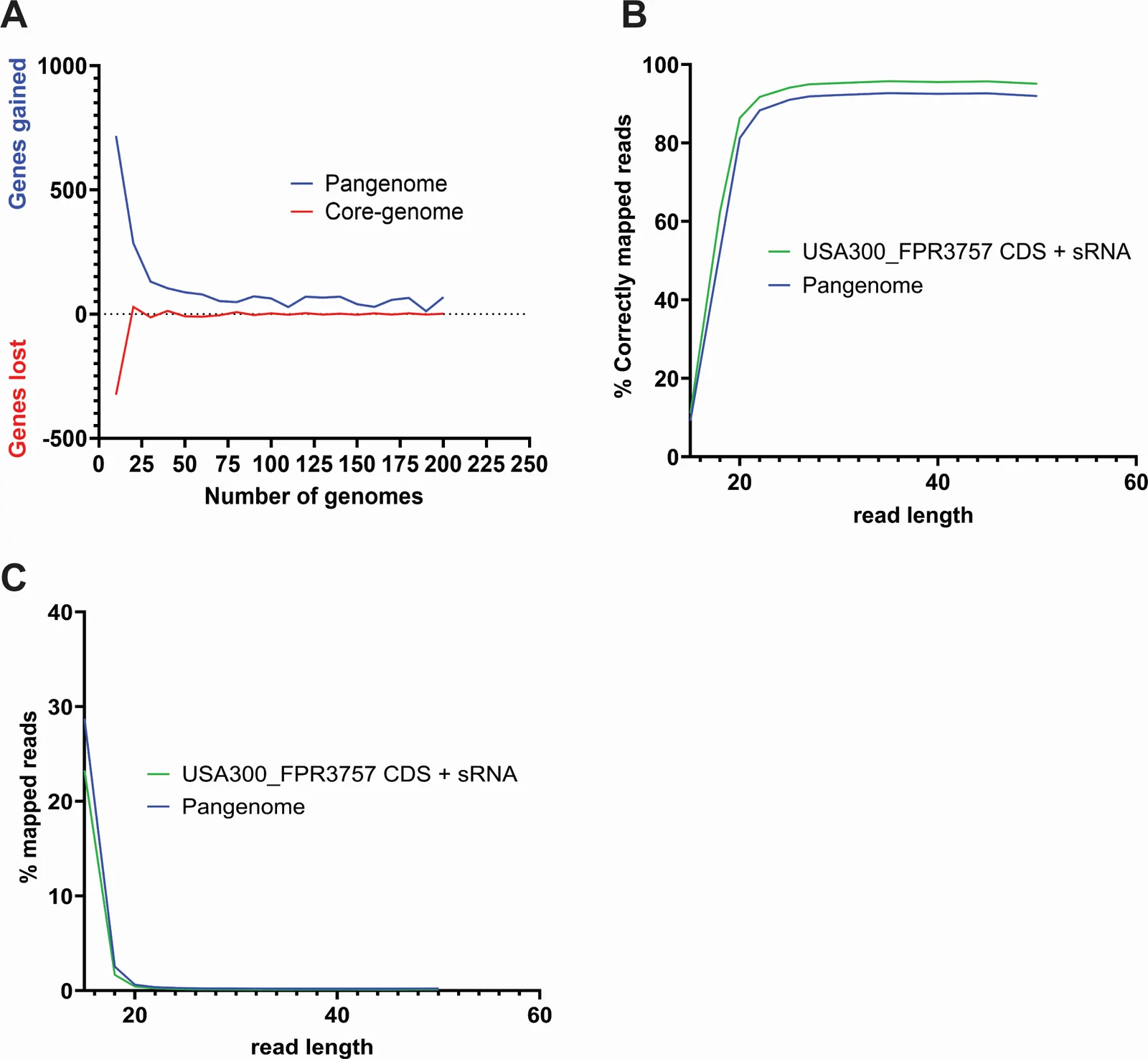
*S. aureus* pangenome construction using Bactopia. (**A**) Rarefaction analysis of the *S. aureus* pangenome was performed by quantifying the number of newly gained genes (upper panel) or lost genes (lower panel) in the pangenome (blue) and core genome (red). (**B and C**) Mapping accuracy of reads with varying length was assessed using *in silico* metatranscriptomic data generated from 110 different bacterial species including *S. aureus* (**B**) or without *S. aureus* (**C**). Reads were mapped to the pangenome (blue) or the USA300_FPR3757 genome (CDS + sRNA, green).

**Fig 3:**
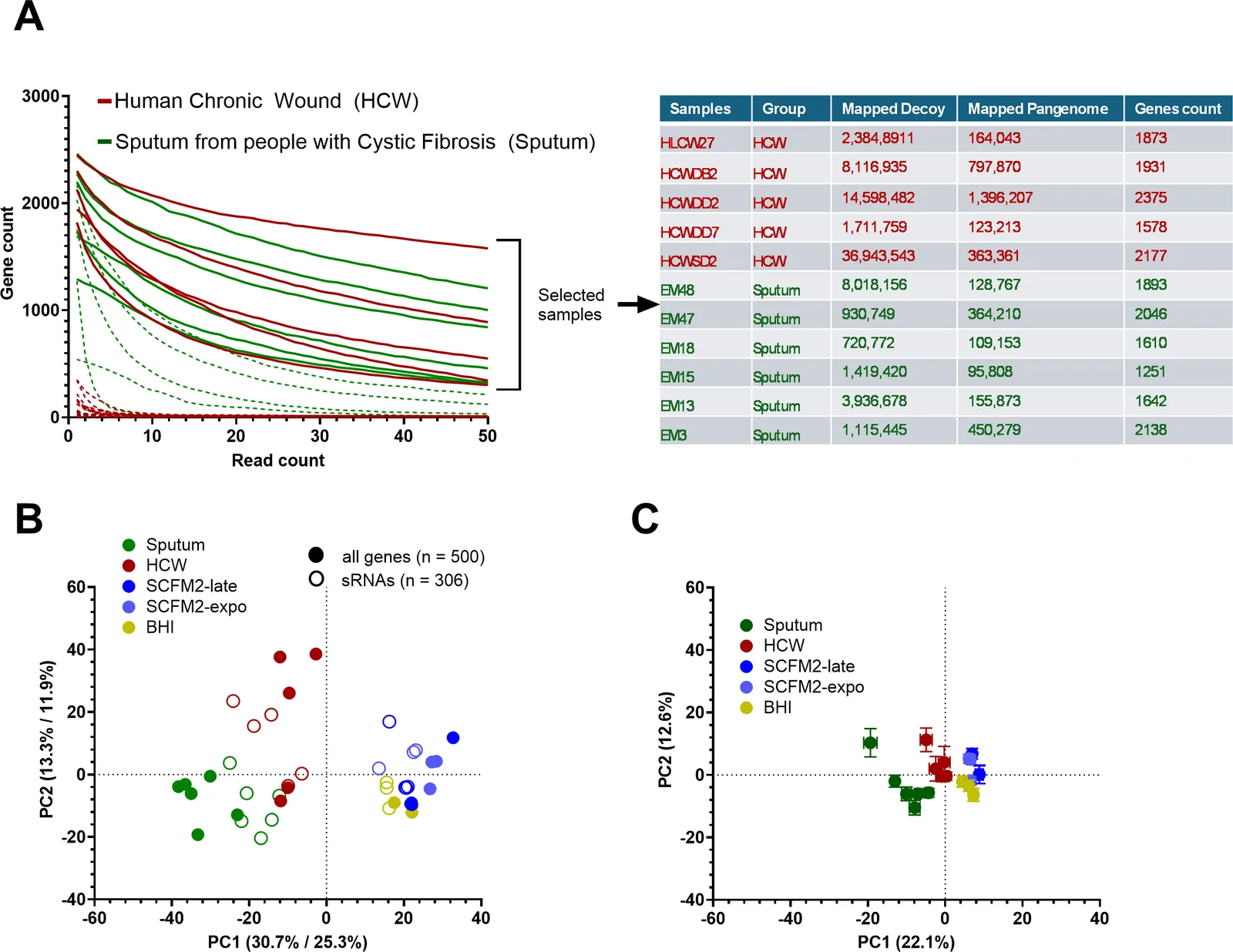
Selection and clustering of *S. aureus* human infection transcriptomes. (**A**) Selection of human chronic infection transcriptomes with high *S. aureus* read counts. The NEMO framework was applied to 43 human chronic wound samples (HCW, red) and 21 sputa from people with cystic fibrosis (sputum, green). Gene read counts were tallied for each sample. Transcriptomes containing reads mapping to at least 1,200 *S. aureus* genes and with at least 300 genes with >50 read counts (solid lines) were retained for downstream analysis. (**B**) The NEMO pipeline was applied to five HCW samples (red), six sputum samples from people with cystic fibrosis (sputum, green), and *S. aureus in vitro* growth controls (BHI, *n* = 3, yellow; SCFM2 during exponential phase, *n* = 4, blue; and SCFM2 during late-exponential phase, *n* = 4). Principal component analysis (PCA) was performed using either 500 genes with the greatest expression variance across samples (CDS + sRNAs; filled circles) or genes encoding sRNAs (open circles). (**C**) To assess whether the separation using sRNAs alone in panel **B** could be explained solely by co-regulation of sRNAs, PCA was performed using 200 bootstrap iterations, each based on a random subset of 306 *S. aureus* genes.

The NEMO workflow comprises three steps (Fig. 1):

- Extraction and sequencing of total RNA from patient-derived infection samples. We used transcriptomes from 43 human chronic wound samples and 21 CF sputum samples. Some of these transcriptomes were previously published by our group (25, 26, 28–31), and others are described here for the first time. To enable direct comparison between *in vivo* and *in vitro* gene expression, we incorporated six *in vitro S. aureus* transcriptomes (25, 26), generated from cells grown in brain heart infusion (BHI) or in synthetic cystic fibrosis sputum medium (SCFM2) (32, 33) (Data set S1).
- Construction of an *S. aureus* pangenome. To construct an *S. aureus* pangenome, we performed a rarefaction analysis using 1,071 publicly available genomes. This analysis indicated that approximately 100 genomes capture the majority of accessory gene diversity, whereas the core genome, defined by orthologous gene clusters present in all strains and sharing ≥99% nucleotide identity, saturates at ~30 genomes (Fig. 2A). Based on these results, we selected 100 genomes for inclusion in pangenome construction and, after excluding two low-quality data sets, used the Bactopia pipeline (34) to retrieve, quality control, assemble, and annotate the remaining 98 high-quality genomes. These included four widely used reference strains (NCTC 8325, N315, USA300_FPR3757, and Newman; Data set S2) and yielded a pangenome comprising 4,386 genes (Data set S3).
- Establishing read length thresholds and accurate mapping to the *S. aureus* pangenome in polymicrobial human infection transcriptomes. Transcriptomic analysis of microbes in human samples is challenging because RNA-seq reads are often short following processing of human infection material. In addition, reads from human infection samples originate from all microbes within these polymicrobial communities. Thus, it is important to ensure accurate mapping of metatranscriptomic reads to the *S. aureus* pangenome. To determine the minimum read length required for accurate mapping, we used an *in silico* approach to define the shortest read length that enables reliable alignment to the *S. aureus* pangenome.
  Mapping sensitivity was evaluated using 11 mock metatranscriptomes generated from 110 reference genomes representing bacterial species commonly associated with chronic human infections, including *S. aureus*. These *in silico* data sets contained reads ranging from 15 to 50 bp and were mapped to either the *S. aureus* reference genome USA300_FPR3757 or the *S. aureus* pangenome. Correct mapping of *S. aureus*-derived reads was limited at 15 bp (10%) but increased sharply at 22 bp, reaching 88.3% for the pangenome and 91.74% for the reference genome (Fig. 2B), identifying 22 nucleotides as the minimum length required for high-confidence mapping. We next evaluated mapping specificity using reads derived exclusively from the 110 reference genomes that did not include *S. aureus*. At 15 bp, 28% and 23% of these reads mapped spuriously to the pangenome and the *S. aureus* reference genome, respectively. In contrast, at 22 bp, incorrect mapping dropped dramatically to 0.37% for the pangenome and 0.25% for the reference genome (Fig. 2C), indicating that 22 nucleotides are also sufficient to minimize cross-species misalignment. This threshold is identical to the minimum read length previously required for accurate mapping to *Porphyromonas gingivalis* in human oral infection metatranscriptomes (31).
  Reads ≥22 nucleotides from human infection metatranscriptomes were subsequently mapped against the decoy database comprising 110 non-*S*. *aureus* species commonly detected in chronic infections. Only reads that failed to align to these decoy genomes were used to map to *S. aureus*, minimizing the risk of incorrectly attributing transcript abundance to *S. aureus* when another species could be responsible. Following mapping, read counts for each gene were normalized using DESeq2 (35), and transcriptomes were retained for downstream analysis based on coverage across the *S. aureus* pangenome, focusing on metatranscriptomes with reads mapping to at least 1,200 genes and containing 300 genes with >50 read counts. Applying this criterion resulted in the selection of transcriptomes from five chronic wound samples and six CF sputum samples for downstream analysis (Fig. 3A). *In silico* typing performed directly from metatranscriptomic reads showed no evidence of active *mecA* expression in any of the 11 analyzed samples. However, because *mecA* expression is inducible and condition dependent (36, 37), this does not definitively exclude the possibility that some isolates are genotypically methicilin-resistant *S. aureus* (MRSA). Multi-locus sequence typing (MLST) yielded a uniquely resolved sequence type for 1 of 11 samples (ST492, clonal complex 1), while partial allelic profiles for two additional samples were found, each consistent with multiple candidate sequence types. *spa* typing resolved 3 of 11 samples (t062 in two chronic wound isolates and t2235 in one CF sputum isolate). Resistance gene profiling, which reflects active transcription of resistance determinants rather than genomic presence alone, identified detectable resistance gene transcripts in 5 of 11 samples. Chronic wound isolates showed a greater number and diversity of expressed resistance genes than CF sputum isolates, including co-transcription of *Aph3-III*, *Ant6-Ia*, and *Sat4A* in two wound samples (Data set S1).

### Identification of sRNAs induced during chronic human infection

Principal component analysis (PCA) based on the 500 *S. aureus* genes with the greatest expression variance across transcriptomes revealed clear separation of human infection samples from *in vitro* controls along the first principal component, which explained 30.7% of the total variance. This indicates a distinct infection-associated transcriptional state (Fig. 3B). Six of the 10 features contributing most strongly to this separation were sRNAs (*RNAIII*, *sprF3*, Teg124, *sprC*, sRNA347, and Sau41; Data set S4). When PCA was restricted to 306 sRNAs expressed in all human infection transcriptomes, similar clustering patterns were observed, with PC1 explaining 25.3% of the variance (Fig. 3B). The distribution of per-gene sRNA expression variance (SD of rlog-normalized counts) was identical across chronic wound, CF sputum, and *in vitro* conditions (Fig. S1). To assess whether this separation could be explained solely by co-regulation of sRNAs, we performed a bootstrap analysis (*n* = 200) using random subsets of 306 *S. aureus* genes. Although samples remained partially grouped by category, both clustering strength and the explained variance were reduced (PC1 = 22.1%), indicating that sRNAs retain discriminatory power beyond general co-regulation and serve as informative markers of human infection-associated transcriptional states (Fig. 3C).

**Fig 4:**
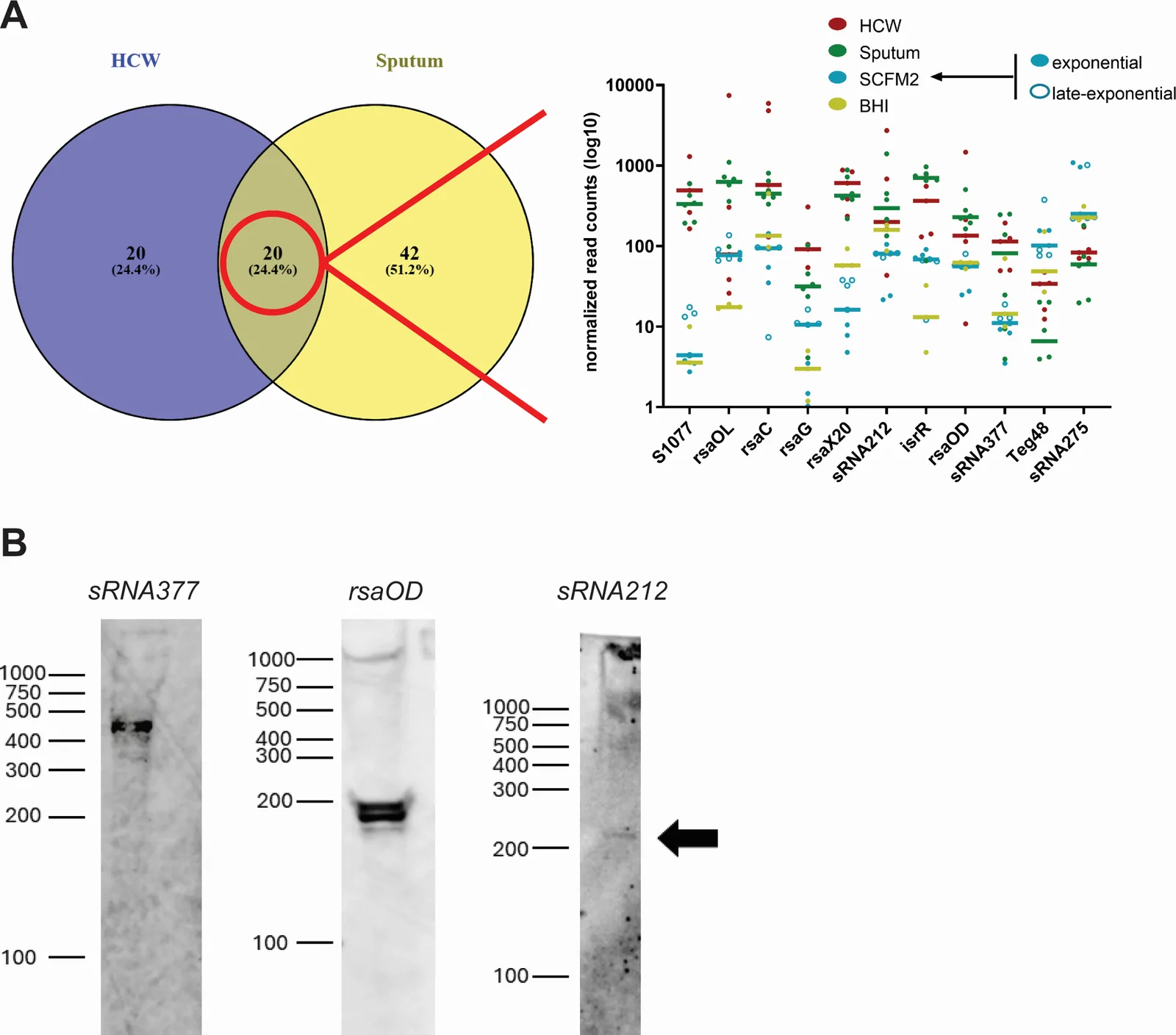
Differential expression analysis reveals a subset of sRNAs highly expressed in human chronic infections. (**A**) Venn diagram of sRNAs induced in human chronic wound (HCW), CF sputum, or both conditions compared to *in vitro* growth using DESeq2. Transcripts with an adjusted *P* <0.05 and |log₂ fold change| >2 were considered differentially expressed. After filtering out lowly expressed sRNAs (<10 normalized read counts), 11 sRNAs (right) were selected for downstream analysis. (**B**) Northern blots of induced sRNAs that have not previously been shown to be bona fide sRNAs.

Next, we performed differential expression analysis to identify sRNAs whose expression differed between human infection samples and *in vitro* growth controls (|log₂ fold change| > 2, adjusted *P* < 0.05). In total, 82 sRNAs showed differential expression, including 20 in human chronic wounds, 42 in CF sputum, and 20 in both infections (Fig. 4A; Data set S5). Because our goal was to identify sRNAs broadly associated with chronic human infection, we focused subsequent analyses on sRNAs differentially expressed in both infection contexts. After filtering out sRNAs with the lowest expression (normalized read count < 10), 11 of the 20 sRNAs were retained. Of these, nine sRNAs, srn_4635_*S1077* (*S1077*), srn_1960_*rsaOL* (*rsaOL*), srn_1590_*rsaC* (*rsaC*), srn_0510_*rsaG* (*rsaG*), srn_4520_*rsaX20* (*rsaX20*), srn_2560_*sRNA212* (*sRNA212*), srn_2975_tsr25 (*IsrR*), srn_3160_*rsaOD* (*rsaOD*), and srn_4610_*sRNA377* (*sRNA377*), showed increased expression during chronic human infection, whereas two sRNAs, srn_1540_*Teg48* (*Teg48*) and srn_3480_*sRNA275* (*sRNA275*), showed lower expression (Fig. 4A).

We chose to focus on sRNAs induced during human infection. Only three of these nine sRNAs have previously been characterized, including *rsaC* and *IsrR* that are involved in metal starvation responses to manganese and iron, respectively (7, 8, 20). The third characterized sRNA is *rsaG*, which is derived from the 3′ untranslated region of the *uhpT* transcript, and responds to glucose-6-phosphate, mucus-secreting cells and contributes to redox homeostasis (16). The remaining six induced sRNAs (*S1077*, *rsaOL*, *rsaX20*, *sRNA212*, *rsaOD*, and *sRNA377*) have not yet been functionally characterized, although *S1077*, *rsaOL*, and *rsaX20* have been previously detected by northern blotting, supporting their classification as bona fide sRNAs (15, 38, 39).

To confirm that the other three genes (*sRNA212*, *rsaOD*, and *sRNA377*) encode bona fide sRNAs, we performed northern blot analysis using RNA extracted from *S. aureus* JE2 grown under rich conditions. Because these sRNAs are weakly expressed *in vitro*, a high RNA input was used to maximize detection sensitivity. Northern blotting confirmed that all three transcripts are expressed as sRNAs (Fig. 4B). Interestingly, *sRNA377* displays a much larger size (~450 nt) than predicted (95 nt), *sRNA212* appears smaller (~210 nt) than expected (~318 nt), and *rsaOD* seems to generate multiple RNA species (ranging from ~180 nt to ~200 nt). Discrepancies between predicted and observed transcript sizes for sRNA377 and sRNA212 likely reflect limitations of the prediction algorithm used for annotation, as well as condition-dependent differences in transcription termination and/or post-transcriptional processing. The multiple species detected for *rsaOD* may similarly result from alternative promoter usage or distinct processing events, both of which have been described for other staphylococcal sRNAs (40, 41). Collectively, these results indicate that *S. aureus* activates expression of nine bona fide sRNAs during chronic human infection.

### Meta-analysis of transcriptomic data identifies distinct sRNA induction patterns

Inducible sRNAs are valuable indicators of the specific environmental and physiological conditions encountered by bacteria during infection, as their expression is often tightly linked to discrete stimuli rather than being constitutively expressed. Leveraging this property across a large repertoire of transcriptomic data sets allows inference of the *in vivo* conditions experienced by *S. aureus* even in the absence of direct environmental measurements. To examine the expression patterns of the nine sRNAs induced during chronic infection, we performed a comprehensive meta-analysis of publicly available *S. aureus* transcriptomic data sets. In total, 2,898 RNA-seq data sets spanning 206 studies were downloaded and processed using NEMO. Runs with fewer than 0.5 million reads mapping to the *S. aureus* pangenome were excluded. After variance-stabilizing transformation (VST) normalization with DESeq2, PCA was used to identify and remove outlier transcriptomes that deviated strongly from the bulk of the data set (Fig. S2). Following re-normalization, the final data set comprised 2,177 transcriptomes from 183 independent studies (Data set S6).

For each of the nine sRNAs, gene-wise *Z*-scores were calculated across all transcriptomes to identify conditions (from transcriptome metadata) that impact expression. *Z*-scores were used as they enable comparison of relative expression levels across large numbers of transcriptomes. Positive *Z*-scores indicate expression higher than the gene’s average across samples, whereas negative *Z*-scores indicate lower-than-average expression. To define thresholds for sRNA induction, we used established sRNA-condition pairs known to induce specific transcripts as internal controls, including *IsrR* in a *fur* mutant, *rsaC* after calprotectin exposure, *sprG2/sprC* in a *sarA* mutant background, and *rsaG* under excess glucose conditions. Based on these controls, each sRNA was classified as induced (*Z*-score > mean control − 2 standard deviations [SD]) or non-induced (*Z*-score < mean control − 2 SD) in each of the 2,177 transcriptomes. Statistical significance was assessed by comparison to non-induced reference samples with multiple-testing correction.

**Fig 5:**
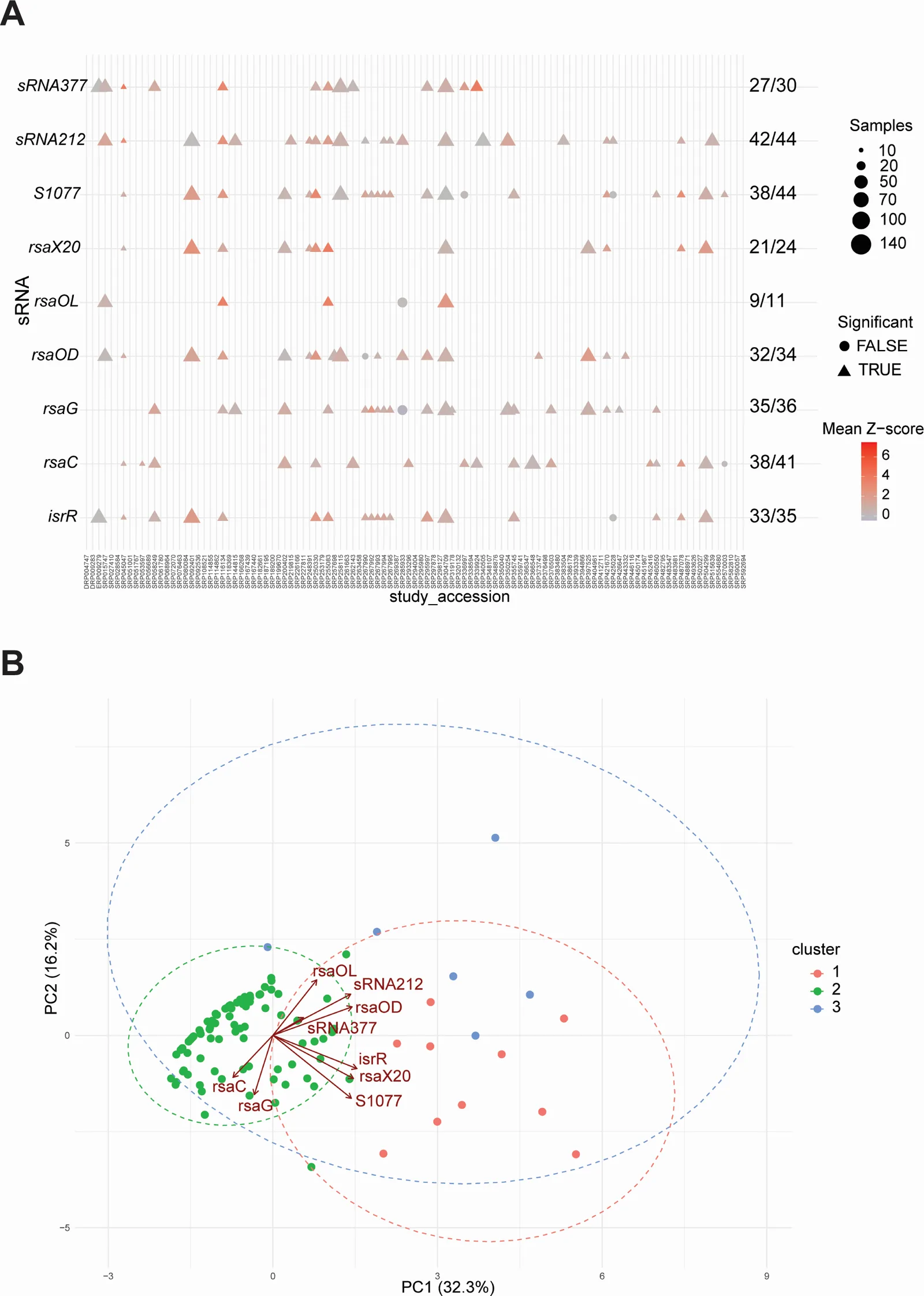
Metatranscriptomic analysis of nine *S*. *aureus* sRNAs induced in human chronic infections. (**A**) Dot plot summarizing induction of selected *S. aureus* sRNAs across publicly available transcriptomic studies. Each point represents one study–sRNA pair. Point color indicates the mean *Z*-score of sRNA expression in the study (gray = lower than global mean; red = higher than global mean). Point size reflects the number of samples included in the study. Point shape denotes statistical significance of induction relative to non-induced reference samples (triangle: adjusted *P* < 0.05; circle: not significant). (**B**) PCA of mean *Z*-scores. Each point represents one transcriptome positioned according to its scores on the first two principal components. Studies are colored according to k-means cluster assignment, and dashed ellipses indicate the 95% confidence regions for each cluster. Red arrows correspond to sRNA loading vectors projected onto the PCA space. Arrow direction indicates the orientation of maximal contribution of each sRNA to principal component separation, while arrow length reflects the magnitude of its contribution.

Visualization of transcriptome-sRNA associations revealed that all nine sRNAs were recurrently induced across multiple independent studies (Fig. 5A). Most sRNAs showed induction in less than 50 of the 2,177 data sets (between 21 and 42), with *rsaOL* induced in the fewest number of samples (nine total). Notably, *rsaX20*/*S1077* and *IsrR* exhibited overlapping induction patterns across studies involving exposure to neutrophils, calprotectin, and chemically defined media, as well as in regulatory mutant backgrounds (e.g., *zur* and *fur* mutants), consistent with shared responses to low-metal conditions (Fig. 5A; Data set S6). To determine whether the chronic wound and CF sputum transcriptomes reflect a metal-starvation signature, we compared gene expression changes in our data sets to those induced by *S. aureus* exposure to calprotectin *in vitro* (42). Gene expression changes were significantly positively correlated between the calprotectin data set and both CF sputum (Spearman *r* = 0.60, 95% CI 0.50–0.68, *P* < 0.0001, *n* = 212) and chronic wound (r = 0.25, 95% CI 0.14–0.36, *P* < 0.0001, *n* = 287) transcriptomes (Fig. S3), consistent with host-imposed nutritional immunity.

To further explore potential co-regulation, we performed PCA on study-level mean *Z*-scores and projected sRNA loadings onto the resulting PCA space (Fig. 5B). K-means clustering grouped studies into three distinct clusters with shared sRNA expression patterns. Cluster 1 (*n* = 10) was enriched for studies using chemically defined media or exposure to stressors such as neutrophils and chemical agents. Cluster 2 (*n* = 86) included studies focused on biofilm formation, polymicrobial interactions, host cell exposure, and antibiotic treatment. Cluster 3 (*n* = 6) included studies examining *S. aureus* under various growth conditions (coculture, T-box riboswitches mutations; Data set S6). Projection of sRNA loadings revealed that *rsaC* and *rsaG* clustered together toward cluster 2, *rsaOL*, *sRNA212*, *sRNA377*, and *rsaOD* toward cluster 3, and *IsrR*, *rsaX20*, and *S1077* toward cluster 1 (Fig. 5B). Together, these patterns indicate that shared experimental conditions across diverse studies are associated with coordinated induction of distinct subsets of sRNAs, consistent with context-dependent co-expression and potential co-regulation.

### *rsaX20* is a zinc-regulated sRNA that also encodes for a small peptide

**Fig 6:**
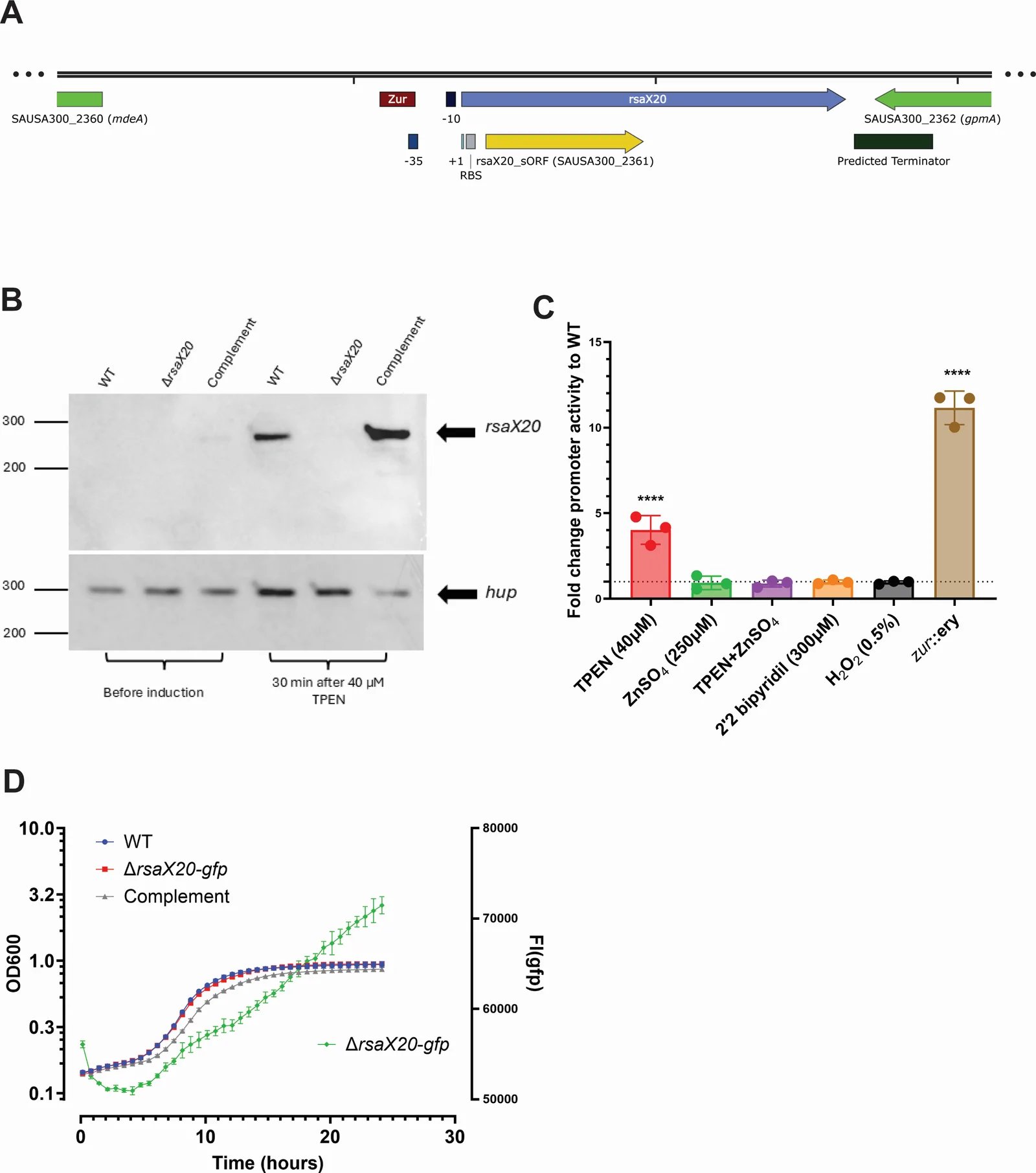
*rsaX20* is a zinc-regulated sRNA that also encodes a small peptide. (**A**) Genomic organization of *rsaX20* (blue) and its neighboring genes (green). The predicted −10 and −35 promoter elements of *rsaX20* are indicated (black and dark blue), together with the transcription start site (+1, gray), the putative small open reading frame (sORF) coding sequence (yellow), and the putative Rho-independent terminator sequence (dark green). (**B**) Northern blot analysis of *rsaX20* expression under zinc-limited conditions. *S. aureus* WT carrying pCN51 (WT), Δ*rsaX20* carrying pCN51 (Δ*rsaX20*), and Δ*rsaX20* carrying pCN51_*rsaX20* (complement) were grown in tryptic soy broth (TSB) with N,N,N′,N′-tetrakis(2-pyridinylmethyl)-1,2-ethanediamine (+TPEN) or without (−TPEN) the zinc chelator TPEN. (**C**) *rsaX20* transcription is induced under zinc limitation and repressed by the zinc-responsive regulator Zur. Cells harboring an *rsaX20::lacZ* transcriptional fusion were grown in TSB or TSB supplemented with TPEN, with or without zinc (ZnSO_4_), as well as under low-iron conditions (2,2′-bipyridyl), oxidative stress (H_2_O_2_), or in a *zur* insertional mutant (*zur::ery*). Promoter activity was quantified by β-galactosidase assays. (**D**) The sORF within the *rsaX20* locus was replaced with green fluorescent protein (*gfp*), and fluorescence was monitored over 24 h in TSB supplemented with TPEN. Statistical significance was assessed using one-way ANOVA with Dunnett’s multiple-comparison test; **** adjusted *P* < 0.0001.

We focused subsequent analyses on *rsaX20*, as transcriptomic data provided clues to its transcriptional regulation, and this locus also contains a predicted sORF encoding a 34-amino acid peptide, raising the possibility that *rsaX20* functions as a dual-purpose sRNA and peptide-coding transcript (Fig. 6A). *rsaX20* is a 255-nt RNA located 238 bp upstream of *mdeA* (SAUSA300_2360), a putative multidrug transporter, and 19 bp downstream of *gpmA* (SAUSA300_2362), which encodes phosphoglycerate mutase (Fig. 6A). A predicted Zur-binding site overlaps the −35 promoter element, consistent with zinc-dependent regulation. Secondary RNA structure prediction using ViennaRNA (43) identified a putative stem–loop seed region containing a UCCCU motif, predicted to base pair with ribosome-binding sites of target mRNAs (Fig. S4)

To confirm zinc-responsive regulation, we performed northern blot analysis in WT *S. aureus* JE2, an *rsaX20* deletion mutant (Δ*rsaX20*), and a genetically complemented strain. Addition of the zinc chelator N,N,N′,N′-tetrakis(2-pyridinylmethyl)-1,2-ethanediamine (TPEN) to the growth media resulted in strong induction of *rsaX20* at the expected size in both wild-type (WT) and complemented strains, indicating induction under zinc-limited conditions (Fig. 6B). This data were confirmed using an *rsaX20::lacZ* transcriptional fusion, which demonstrated a fourfold increase in *rsaX20* promoter activity under TPEN treatment, which was fully reversed by zinc supplementation, while neither iron limitation nor oxidative stress induced expression (Fig. 6C). Mutation of the gene encoding the transcription factor Zur led to strong promoter activation, confirming that Zur represses *rsaX20* under zinc-replete conditions (Fig. 6C). These data indicate that *rsaX20* is controlled by the Zur repressor and inducible by low zinc conditions.

To assess whether the *rsaX20* transcript is translated, the predicted *rsaX20* sORF was replaced chromosomally with the green fluorescence protein (GFP), while retaining the native ribosome-binding site (strain referred to as Δ*rsaX20-gfp*). Under zinc-limiting conditions, GFP fluorescence increased beginning in exponential phase (~5 h) with no detectable growth defects, indicating active translation from the *rsaX20* transcript (Fig. 6D). In further support of the *rsaX20* sRNA encoding a peptide, the native *rsaX20* peptide was also observed via proteomics of TPEN-exposed *S. aureus* (described in detail below).

Together, these results demonstrate that *rsaX20* encodes a zinc-inducible sRNA that encodes a small open reading frame, suggesting that it may function as a dual-purpose sRNA and peptide.

### Proteomic, transcriptomic, and genetic analyses support a dual sRNA/sORF role for *rsaX20* in zinc limitation

To further investigate the functional role of *rsaX20* under zinc-limiting conditions, we performed transcriptomic and proteomic analyses of WT, Δ*rsaX20*, and genetically complemented Δ*rsaX20* grown in tryptic soy broth (TSB) supplemented with TPEN (Data sets S7 and S8). Initial characterization of the Δ*rsaX20* deletion mutant revealed a polar effect on the downstream gene *mdeA*, with *mdeA* expression elevated in both the mutant and the complementation strain relative to wild type, indicating that this effect was not rescued by extrachromosomal complementation (Fig. S5A). To determine whether this effect also occurred in Δ*rsaX20-gfp*, we quantified *mdeA* expression by RT-qPCR. In contrast to the deletion mutant, *mdeA* expression in Δ*rsaX20-gfp* was comparable to WT levels (Fig. S5B), indicating that the polar effect was specific to Δ*rsaX20*.

Based on these results, we performed additional proteomics experiments using ∆*rsaX20-gfp* and a strain carrying a chromosomal start-codon mutation in the *rsaX20* sORF (sORF*). These strains eliminated the impact of polar effects caused by *rsaX20* deletion and enabled separation of regulatory effects attributable to the *rsaX20* sRNA vs the peptide. Both cytoplasmic proteins and secreted proteins were analyzed to capture a broad spectrum of *rsaX20*-dependent regulation under zinc limitation. Protein abundance changes attributable to *rsaX20* were quantified as log_2_ ratios for: (i) WT vs ∆*rsaX20-gfp*; (ii) WT vs ∆*rsaX20-gfp* complement strain (expressing both the sRNA and peptide); (iii) WT vs sORF* (expressing only the sRNA); and (iv) WT vs ∆*rsaX20-gfp* complemented only with the sRNA (not the peptide). Along with proteomics, RNA-seq was also used to assess whether observed protein-level changes could be explained by transcriptional effects.

**Fig 7:**
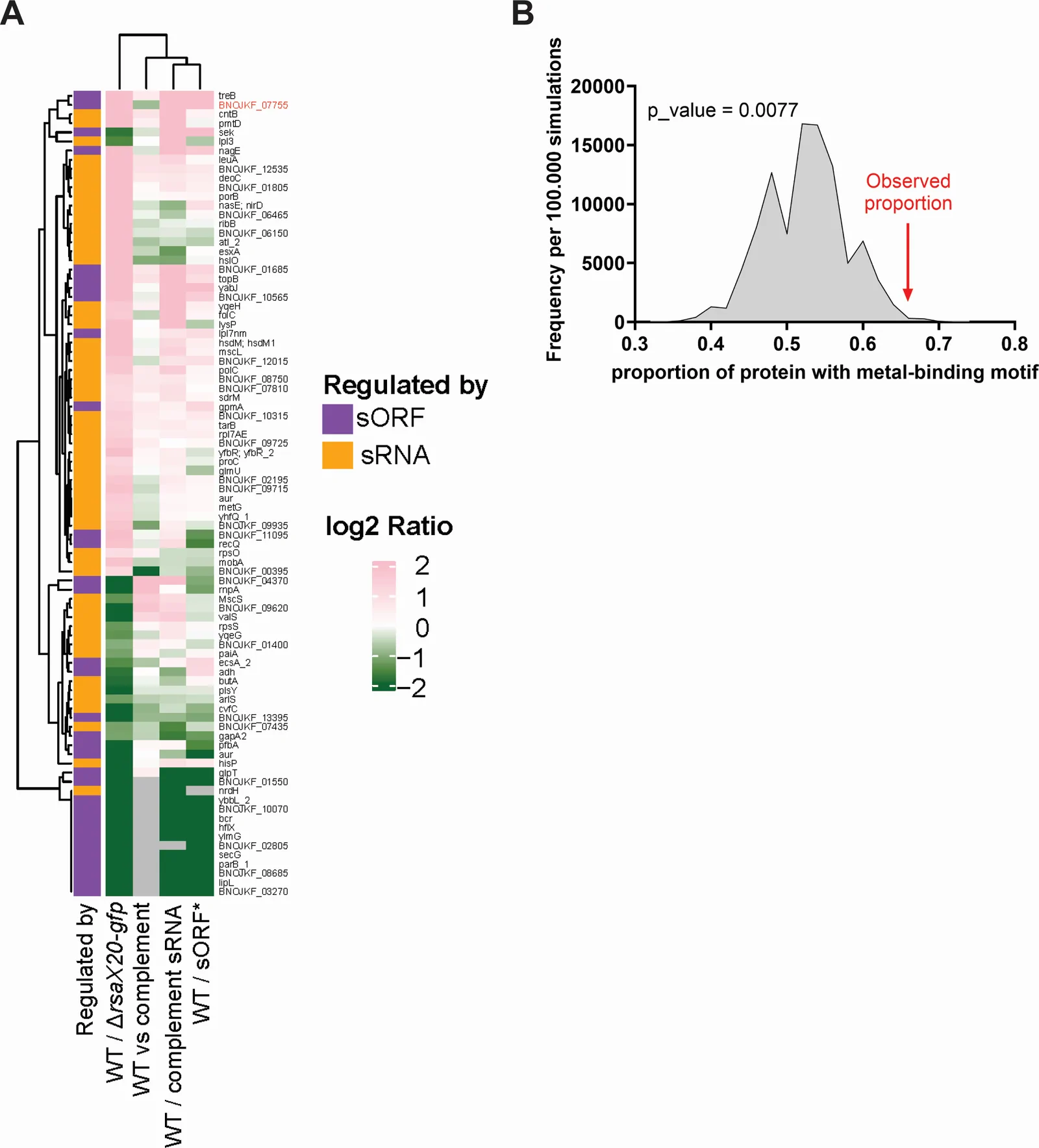
Proteomics, transcriptomics, and metal-binding profiling of *rsaX20*-regulated proteins under low-zinc conditions. (**A**) *S. aureus* WT + pCN51 (WT), ∆*rsaX20-gfp* + pCN51, ∆*rsaX20-gfp* + pCN51_*rsaX20* (complement), *S. aureus* containing a chromosomal point mutation in the sORF start codon (sORF*), and ∆*rsaX20-gfp* + pCN51_*rsaX20*_sORF* (complement sRNA) were grown in TSB supplemented with 40 µM TPEN to exponential phase. Total proteins were extracted and subjected to quantitative proteomics analysis. The heatmap shows log_2_ protein abundance ratios across four pairwise comparisons: WT vs ∆*rsaX20-gfp* (both sRNA and sORF disrupted), WT vs complement (∆*rsaX20-gfp* complemented with both the sRNA and sORF strain), WT vs complement sRNA (∆*rsaX20-gfp* complemented with only the sRNA), and WT vs sORF* (chromosomal mutation of the sORF start codon). Protein abundance values were log_2_ transformed and hierarchically clustered by rows and columns. Proteins are classified by which regulator (sRNA or sORF) regulates them. Red label indicates the *rsaX20* sORF. (**B**) Distribution of metal-binding protein proportions (determined by motif scanning) obtained by randomly sampling 88 proteins from the *S. aureus* proteome 100,000 times (Monte Carlo simulation). The red arrow indicates the observed proportion in the *rsaX20* targets (65.9%). Enrichment of metal-binding proteins was assessed using a permutation test.

In total, 79 cytoplasmic and 9 secreted proteins were differentially abundant (|log₂ ratio| > 2, adjusted *P* < 0.05; Fig. 7A; Data set S9). As mentioned above, proteomic data confirmed translation of the *rsaX20* sORF, and the start-codon mutation eliminated detectable peptide production (WT vs sORF* abundance ratio >100). Overall, 33 proteins were predicted to be regulated by the sORF and 55 by the *rsaX20* sRNA (Fig. 7A). sRNA-regulated proteins were significantly more likely to be positively regulated than sORF-regulated proteins (Fisher’s exact test, *P* = 0.0015; odds ratio = 4.57), consistent with opposing regulatory roles. Notably, only two protein-level changes could be attributed to corresponding transcriptional differences, underscoring the predominant post-transcriptional nature of *rsaX20*-mediated regulation (Data set S9).

Among the sRNA-repressed targets were aureolysin (Aur) and alcohol dehydrogenase (AdhP), both of which require zinc as a cofactor, consistent with a zinc-sparing response. Motif analysis of all 88 proteins controlled by the *rsaX20* sRNA or protein revealed significant enrichment for metal-binding–permissive motifs, including DxH/HxD, HxH, CxxC, HxxH, and Fe–S cluster motifs (Fig. 7B; Fig. S6; Data set S9). Overall, 58 of the 88 (65.9%) target proteins contained at least one such motif, significantly higher than expected by chance based on Monte Carlo resampling of *S. aureus* protein-encoding genes (100,000 iterations; *P* value = 0.0077), indicating preferential regulation of metal-associated proteins under zinc limitation. Translational fusions of *lacZ* to *adhP* and *paiA* validated our proteomics data (Fig. S7). Collectively, these data support a model in which *rsaX20* functions as a dual sRNA/sORF regulator that modulates metal-associated proteins during zinc limitation (44).

## DISCUSSION

Understanding which bacterial genes are expressed during human infection is critical, as *in vitro* and *in vivo* models often fail to capture the complexity of the human infection environment (25, 28, 31). Our group has previously generated high-coverage transcriptomes from multiple bacterial species during human infection (25–27), producing publicly available sputum and chronic wound data sets that provide a valuable resource for studying *in vivo* gene expression. In the present work, we expanded these data sets and focused on *S. aureus* sRNA expression during chronic infection, a regulatory layer that remains largely underrepresented in most transcriptomic analyses despite its central role in gene regulation. This was accomplished through the development and application of NEMO to *S. aureus* using a curated pangenome framework. We identified that a minimum read length of 22 nucleotides is required for reliable mapping of *S. aureus* reads, a threshold that mirrors previous findings in *P. gingivalis* (31), suggesting that shared constraints on informative read length may exist across bacterial species.

Unsupervised clustering revealed that sRNAs contribute disproportionately to transcriptional variability during chronic human infection. Six of the 10 strongest variance-driving transcriptome features were sRNAs, with *RNAIII* ranked highest. When expanded to the top 100 features, sRNAs accounted for nearly one-third of the total. *RNAIII*, the central effector of the *S. aureus* quorum-sensing system (12, 45–48), was consistently repressed in chronic infection samples, particularly in CF sputum. This pattern supports a shift toward a less virulent transcriptional state during chronic human infection, consistent with prior observations in animal models showing reduced toxin expression during colonization-like states (49). Although broader sampling of chronic infection contexts will be required to thoroughly test this hypothesis, the strong representation of sRNAs among variance-driving features suggests their importance in shaping the outcome of human infections.

A central outcome of this study was the identification of 82 sRNAs differentially expressed during chronic human infection relative to *in vitro* conditions, the majority of which have not been previously characterized (7, 8, 12, 16, 19, 20, 23, 40, 50–58). Notably, most of this chronic-infection-associated sRNA response was niche specific: 20 of these 82 sRNAs (24.4%) were differentially expressed in both chronic wounds and CF sputum, while 20 were specific to wounds and 42 specific to sputum, highlighting divergence in the *S. aureus* transcriptional response across distinct chronic infection environments. In chronic wounds, the most strongly induced sRNAs belonged to a well-described type I toxin–antitoxin system, consistent with repression of toxin production. In contrast, the most highly induced sRNAs in sputum samples, *Sau63* and *Teg107*, remain uncharacterized, underscoring the limited understanding of sRNAs most abundantly expressed during human infection. This pronounced niche specificity may reflect distinct local host environments, polymicrobial community composition, or site-specific selective pressures, representing a promising avenue for future investigation into infection-site-specific *S. aureus* adaptation.

Among sRNAs induced in both wound and sputum samples, only *rsaC*, *rsaG*, and *IsrR* have been previously described. *rsaC* and *IsrR* mediate manganese- and iron-sparing responses, respectively, providing internal validation for our approach and supporting the presence of nutritional immunity in human infection sites (26, 31, 59). Extending this paradigm, our analyses identified *rsaX20* as a zinc-responsive sRNA regulated by Zur. Large-scale transcriptomic meta-analysis across 183 studies confirmed recurrent induction of *rsaX20* under low-metal conditions, while also revealing context-specific expression patterns for other sRNAs.

Proteomic analyses further showed that *rsaX20* functions as a dual sRNA/sORF regulator targeting proteins enriched in metal-binding motifs. Notably, *rsaX20* represses the zinc-dependent AdhP, consistent with a zinc-sparing response, although the physiological consequences of this repression remain unclear. While *rsaX20*-mediated base pairing regulates AdhP translation (44), the function of the encoded peptide remains unresolved, and *rsaX20* appears dispensable for growth under zinc limitation (Fig. 6D), in contrast to *IsrR* under iron starvation (7). This difference may reflect lower zinc essentiality or redundancy among zinc-sparing mechanisms, potentially including additional sRNAs such as *S1077*, which originates from the 5′ UTR of the *cnt* operon (60). Interestingly, *S. aureus* genes classified as essential using a mouse infection model (61) were more frequently positively regulated by the *rsaX20* sRNA, suggesting a potential contribution to *S. aureus* fitness during infection (Data set S9). Zinc starvation imposed by host nutritional immunity has substantial consequences for *S. aureus* physiology and pathogenesis. Zinc serves as a cofactor for a large number of bacterial proteins, including enzymes involved in central metabolism, DNA replication, and oxidative stress defense (3, 4). To counteract host-imposed zinc limitation, *S. aureus* uses two zinc import systems, AdcABC and the staphylopine-dependent CntABCDF system. These two systems are functionally redundant for virulence: single mutants in either pathway show minimal reduced virulence in mice, but combined loss of both systems significantly impairs *S. aureus* infection *in vivo*, demonstrating that zinc acquisition is essential for pathogenesis and that *S. aureus* maintains redundant mechanisms to ensure it (62). Our identification of *rsaX20* as a Zur-regulated sRNA induced under zinc-limiting conditions encountered in chronic wound and CF sputum infection adds a previously uncharacterized layer to this regulatory network, suggesting that *S. aureus* deploys a coordinated transcriptional response to nutritional immunity that extends beyond canonical import systems and metalloproteins to include small regulatory RNAs.

While the function of the *rsaX20*-encoded peptide remains undefined, several bacterial sRNAs encode both a regulatory RNA and a functional peptide, demonstrating that a single locus can exert independent effects on gene expression at multiple levels. For example, the *E. coli* sRNA *SgrS* represses target mRNAs through base pairing while also encoding the small protein SgrT, which independently modulates glucose transport and cellular physiology (63). Our data indicate that *rsaX20* independently alters the abundance of a subset of proteins, suggesting that this locus similarly integrates classical sRNA regulation with peptide-mediated control. One plausible model is that *rsaX20* influences zinc-dependent processes or metal allocation, similar to the *E. coli* small peptide MntS (64, 65), thereby reshaping protein abundance under zinc limitation. Determining the biochemical mechanism of *rsaX20* will be essential for understanding how these two regulatory activities are coordinated.

Although our data support a regulatory link between *rsaX20* and aureolysin, an established *S. aureus* virulence factor, we did not observe dysregulation of major toxins or canonical virulence regulators under the conditions examined here. Resolving the precise contribution of *rsaX20* to virulence and persistence will require direct functional testing of *rsaX20* deletion phenotypes, which could include murine infection models among other approaches, alongside continued analysis of human infection transcriptomes as the primary basis for identifying physiologically relevant conditions to test.

In summary, this study used NEMO to systematically characterize *S. aureus* sRNA expression during chronic human infection and identified a zinc-responsive sRNA associated with metal-sparing regulation. Whether the regulatory features identified here, including zinc-responsive sRNA *rsaX20*, are specific to chronic infection or represent a broader adaptive strategy employed by *S. aureus* across infection types, including acute infections, remains an open question. Future work directly comparing sRNA expression patterns across acute and chronic infection contexts will be necessary to resolve this distinction. Together, these findings broaden our understanding of metal homeostasis and sRNA-mediated regulation in *S. aureus* and emphasize the importance of incorporating sRNAs into metatranscriptomic analyses of bacterial infections.

## MATERIALS AND METHODS

### Strains and growth conditions

All bacterial strains, oligonucleotides, and plasmids used in this study are listed in Table S1. *Staphylococcus aureus* strains were routinely streaked onto tryptic soy agar (TSA) plates and grown in TSB at 37°C with shaking (200 rpm) for 6 h, supplemented with antibiotics when indicated. *Escherichia coli* strains were grown in Luria–Bertani (LB) medium at 37°C with shaking.

For induction experiments, cultures grown in TSB were supplemented with the zinc chelator N,N,N′,N′-tetrakis(2-pyridinylmethyl)-1,2-ethanediamine (TPEN; 40 µM), the iron chelator 2,2′-bipyridyl (250 µM), hydrogen peroxide (0.5%), or zinc sulfate (ZnSO_4_, 250 µM). Fluorescence experiments were performed in 96-well plates with a final volume of 200 µL per well using an Agilent BioTek Synergy H1 multimode reader set at 37°C with double orbital shaking.

Clean deletion of *rsaX20* (Δ*rsaX20*), construction of the *rsaX20* GFP insertion strain (Δ*rsaX20-gfp*), and generation of the *rsaX20* stop-codon mutant (sORF*) were performed by allelic exchange using the plasmid pIMAY-Z (66). Briefly, DNA fragments encompassing 1 kb upstream and downstream of the *rsaX20* locus, a 2 kb fragment of the *rsaX20* locus with the *S. aureus* codon-optimized *gfp* replacing the sORF *rsaX20* sequence, and a 2 kb fragment of the *rsaX20* locus containing a stop codon (TAG) were synthesized by Twist Bioscience. These fragments were PCR-amplified using oligonucleotides OPRB25/26 and cloned into PCR-linearized pIMAY-Z (OPRB11/12) by *in vivo* assembly (67) in *E. coli* IM08B competent cells. Validated plasmids were transformed into *S. aureus* JE2 (68), and double-crossover recombinants were selected. Candidate mutants were confirmed by whole-genome sequencing (Plasmidsaurus).

For complementation, the *rsaX20* locus (200 bp upstream of the transcription start site and 100 bp downstream of the terminator) was PCR amplified using oligonucleotides OPRB116/117, double-digested with *BamHI* and *EcoRI*, and ligated into the pCN51 vector (69). The ligation mixture was transformed into *E. coli* IM08B, and plasmids were extracted and sequence verified. Confirmed plasmids were then introduced into *S. aureus* JE2 Δ*rsaX20* and ∆*rsaX20-gfp* strains and selected on TSA supplemented with erythromycin (5 µg/mL). To generate the *rsaX20* sRNA complement strain, genomic DNA from the sORF* strain was used as a template with oligonucleotides OPRB116/117 and cloned into pCN51 as described above.

The pJB185 vector (70) was used for β-galactosidase assays. The *rsaX20* promoter was PCR-amplified using oligonucleotides OPRB85/86, double-digested with *EcoRI* and *BamHI*, and ligated into pJB185. Following transformation into *E. coli* IM08B and selection on LB agar supplemented with ampicillin, plasmids were extracted and sequence-verified before being transferred into *S. aureus* JE2 WT competent cells.

For translational fusion assays, 250 bp of the native promoter region and the first 99 nucleotides of the corresponding coding sequence were (*paiA* and *adhP*) synthesized by Twist Bioscience and inserted into the *XbaI* site of pJB185. All constructs were initially transformed into the restriction-modification-deficient strain *S. aureus* RN4220 (23) and subsequently phage-transduced using bacteriophage Φ11 into the final recipient strains (JE2 WT, ∆*rsaX20-gfp*, or sORF*).

### RNA extraction and sequencing

Human chronic wound samples were processed as previously described (25). Samples were immediately preserved in RNAlater after collection and stored at 4°C overnight prior to storage at −80°C. Samples were thawed on ice, centrifuged at 10,000 × *g* for 30 min at 4°C to remove RNAlater, and transferred to bead-beating tubes containing a mixture of 2 mm zirconia and 0.1 mm zirconia–silica beads. Samples were resuspended in RNase- and DNase-free TE buffer and enzymatically lysed with lysozyme (1 mg/mL final concentration) and lysostaphin (0.17 mg/mL final concentration) at 37°C for 30 min. Mechanical lysis was performed by bead beating three times for 30 s while cooling on ice between cycles. Total RNA was extracted using RNA-Stat 60 followed by chloroform phase separation (200 μL chloroform per 1 mL RNA-Stat 60) and centrifugation at 12,000 × *g* for 15 min at 4°C. The aqueous phase was precipitated with isopropanol (0.5 mL per 1 mL RNA-Bee) in the presence of 20 μg linear acrylamide and incubated at −80°C overnight. RNA pellets were recovered by centrifugation at 12,000 × *g* for 30 min at 4°C, washed with 75% ethanol, air-dried, and resuspended in RNase-free water. RNA concentration was determined by NanoDrop spectrophotometer, and quality was confirmed by Bioanalyzer. Ribosomal RNA (rRNA) was depleted using the RiboZero Gold bacterial kit for *in vitro* samples and the RiboZero Gold epidemiology kit for human samples, followed by ethanol precipitation. Depleted RNA was fragmented for 2 min using the NEBNext Magnesium RNA Fragmentation Module, and cDNA libraries were prepared using the NEBNext Multiplex Small RNA Library Prep Kit according to the manufacturer’s instructions. Libraries were sequenced on an Illumina NextSeq 500 platform using 75 bp single-end reads.

### RNA-seq data processing and analysis pipeline

Read retrieval sequencing reads were downloaded from the NCBI Sequence Read Archive using prefetch, followed by conversion to FASTQ format with fasterq-dump. Single-end and paired-end libraries were handled separately to ensure appropriate downstream processing. FASTQ files generated at this stage were used as input for trimming. Adapter trimming was performed using Trim Galore (v0.6.x), which internally uses Cutadapt for adapter removal. Trimming was applied separately to single-end and paired-end libraries. Adapter sequences were automatically detected based on standard Illumina adapters. Reads shorter than 22 nucleotides after trimming were discarded. For paired-end libraries, trimming was performed in paired mode, and read pairs were retained only if both mates passed trimming and length filtering. To remove residual rRNA reads, trimmed reads were aligned against an rRNA reference database using Bowtie2. Following rRNA filtering, reads were aligned to a decoy reference genome using Bowtie2 to eliminate non-*S*. *aureus* reads. Unmapped reads were aligned to a *S. aureus* pangenome reference using Bowtie2. Single-end and paired-end libraries were aligned using the appropriate Bowtie2 modes. Strand specificity was inferred independently for each sample prior to final quantification. Final gene-level read counts were generated using featureCounts with the inferred strand setting for each sample.

### Pangenome construction and analysis

To generate a comprehensive pangenome for *S. aureus*, we used the Bactopia Tools pangenome workflow (v ≥ 2.2.0) with default settings, employing PIRATE for core and accessory gene clustering (https://github.com/bactopia/bactopia) (34). A total of 100 *S. aureus* genomes were included in the analysis. This set comprised 96 assembled genomes of clinical and lab isolates together with four reference genomes (*Staphylococcus aureus* NCTC 8325, N315, USA300_FPR3757, and Newman). All steps were executed with default filtering and clustering parameters. After the generation of the pangenome FASTA sequence and corresponding gene feature format (GFF) files, we retrieved the sequences of 651 unique sRNAs annotated in the Staphylococcal Regulatory Database (SRD; https://srd.genouest.org/) across all available *S. aureus* species. Each sRNA sequence was aligned to the pangenome using BLAST to identify intragenic sRNAs; their genomic coordinates and strand orientation were recorded, and the GFF files were updated accordingly. For sRNAs not mapping intragenically, sequences were appended to the pangenome FASTA file and individually re-aligned against the updated pangenome to determine their genomic location and orientation. These annotations were then incorporated into the final GFF file. To assess mapping efficiency, we classified pangenome genes into four categories. As the pangenome represents the union of core and accessory genes across 98 *S. aureus* genomes, genes were labeled “unique” if they belonged to the accessory genome of one or more constituent strains but were absent from the USA300 FPR3757 reference genome specifically. Genes were labeled “normally expressed” if their expression levels in *in vitro*–grown *S. aureus* (BHI medium) were not significantly different when reads were mapped to the pangenome vs the reference genome. Genes were classified as “mismapped” when expression was detected for the pangenome gene but not for the corresponding gene in the reference genome. Finally, genes were labeled “overexpressed” or “underexpressed” when differential expression analysis revealed significant expression differences between mappings (Data set S3).

#### MLST and resistance gene detection

Multi-locus sequence typing and antimicrobial resistance gene detection were performed using SRST2 v0.2.0 (-min_coverage 80 --max_divergence 10). MLST used the *S. aureus* allele database and sequence type definitions downloaded from PubMLST (https://pubmlst.org/organisms/staphylococcus-aureus) via getmlst.py. Resistance gene detection used the ARG-ANNOT r3 database. For samples where SRST2 returned an incomplete or unresolved sequence type (“NF”), the called partial allelic profile was manually queried against the PubMLST *S. aureus* sequence definition database (https://pubmlst.org/bigsdb?db=pubmlst_saureus_seqdef) to determine whether the available alleles uniquely matched a single sequence type. Profiles that matched more than one sequence type, or that matched none, are reported as not determined.

#### *mecA* expression assessment

*mecA* status was assessed using two complementary read-mapping approaches rather than relying on detection thresholds alone: (i) direct mapping of pangenome-extracted reads to the canonical *mecA*-containing reference sequence (GenBank AB033763) using Bowtie2 (v2.2.8), followed by coverage assessment with samtools coverage; and (ii) direct inspection of read coverage at the *mecA* locus within the *S. aureus* pangenome (coordinates 2,494,132–2,496,138). Samples were considered to show no evidence of active *mecA* expression if both methods yielded negligible coverage and depth.

#### *spa* typing

Reads mapping to the *spa* locus within the pangenome (coordinates 812,650–814,320) were extracted and assembled using SPAdes v4.2.0 in RNA-seq mode (--rna), and the resulting transcripts were typed using spaTyper. Samples for which assembly did not yield a contig matching a recognized repeat pattern are reported as non-typeable.

### Northern blot

Total RNA was extracted using the same protocol as for human samples. RNA integrity was assessed by denaturing TBE-urea gel electrophoresis. Templates for riboprobe synthesis were generated by PCR amplification of the antisense strand of the target RNA, including a T7 promoter sequence at the 5′ end, using Q5 High-Fidelity DNA polymerase (NEB). PCR products were verified on an agarose gel and purified using a commercial PCR cleanup kit (OPRB43/44 for *rsaX20*, OPRB190/191 for *sRNA212*, OPRB187/188 for *rsaOD*, OPRB183/184 for *sRNA377*, and OPRB55/56 for housekeeping gene *hup*). DIG-labeled RNA probes were synthesized using the HiScribe T7 High Yield RNA Synthesis Kit (NEB) with digoxigenin-11-UTP (DIG-11-UTP; Roche) according to the manufacturer’s instructions. Briefly, transcription reactions (20 µL) contained 0.5 µg of PCR template, 1.5 µL each of ATP, CTP, GTP, and UTP (100 mM), 5 µL DIG-11-UTP, 1.5 µL T7 RNA polymerase mix, 1 µL DTT (0.1 M), 1.5 µL 10 × reaction buffer, and nuclease-free water. Reactions were incubated at 37°C for 4–5 h, followed by DNase I treatment (2 µL, 37°C, 15 min). RNA probes were purified using the GeneJET RNA Cleanup and Concentration Micro Kit (ThermoFisher), eluted in nuclease-free water at ≥75 ng/µL, and stored at −20°C until use. RNA samples (5–15 µg) were mixed 1:1 with 2 × RNA loading dye (NEB), denatured at 70°C for 10 min, and immediately chilled on ice. Samples and the Ambion RNA Century-Plus Markers were resolved on 10% Mini-PROTEAN TBE-Urea gels (Bio-Rad) in 1× TBE buffer. Gels were pre-run at 100 V for 45 min, and electrophoresis was performed at 50 V for 10 min, followed by 100 V until the bromophenol blue dye front reached the bottom (~2–3 h). RNA was transferred onto positively charged nylon membranes (Hybond N+, GE Healthcare) using a semi-dry Trans-Blot apparatus (Bio-Rad) in 0.5× TBE buffer at 20 V for 35 min. Membranes were UV crosslinked (1,200 µJ/cm², twice) and stained with 0.02% methylene blue in 0.5 M acetic acid to visualize 5S rRNA and the RNA marker.

Membranes were pre-hybridized in DIG Easy Hyb (Roche) at 68°C for 30 min. Denatured DIG-labeled riboprobes (100 ng/mL) were added, and hybridization was performed overnight at 68°C with gentle rotation. Membranes were washed sequentially in 2× SSC/0.1% SDS (2 × 5 min) and 0.1× SSC/0.1% SDS (2 × 15 min) at room temperature. After equilibration in 1× washing buffer, membranes were blocked for 30 min in 1× blocking solution and incubated with anti-DIG-AP antibody (1:10,000) for 30 min. Following washes in 1× washing buffer (2 × 15 min), chemiluminescent detection was performed using CDP-Star substrate (Roche), and signals were visualized using a G-Box imaging system (Syngene).

### Global metatranscriptomic normalization and *Z*-score analysis

Gene-level raw read counts were obtained from featureCounts output files generated for each RNA-seq sample (using the same pipeline as human samples). Sample metadata, including SRA run accessions and corresponding BioProject identifiers, were imported from a curated metadata table. Only samples for which featureCounts output files were available were retained for downstream analysis. A global raw count matrix was constructed by merging gene-level counts across all samples. Missing values resulting from incomplete gene coverage across samples were replaced with zeros. To remove low-quality libraries, samples with fewer than 500,000 total mapped reads across all genes were excluded from further analysis. Genes with low expression across the data set were filtered to reduce noise. Specifically, genes were retained if they had at least 10 raw counts in a minimum of 375 samples across the full data set. This threshold was chosen following an empirical assessment of gene retention. Filtered raw counts were normalized using DESeq2 to account for differences in library size and sequencing depth across samples. A DESeqDataSet was constructed using BioProject identifiers as a covariate in the design formula to account for study-specific effects during size factor estimation. DESeq2 normalization was performed without differential expression testing, and normalized counts were extracted for downstream analyses. To stabilize variance across the dynamic range of expression values, normalized counts were transformed using the VST implemented in DESeq2. For comparative metatranscriptomic analyses across heterogeneous data sets, per-gene *Z*-scores were computed from the VST-transformed expression matrix. For each gene, expression values were centered and scaled across all samples by subtracting the gene-wise mean and dividing by the gene-wise standard deviation. To assess global expression structure and identify potential batch effects or outliers, PCA was performed on the VST-transformed expression matrix. The top 500 most variable genes, as determined by row-wise variance, were selected for PCA. Potential outlier samples were identified using PCA-based distance metrics. Following outlier detection, DESeq2 normalization, VST transformation, and per-gene *Z*-score computation were repeated using only non-outlier samples.

### β-Galactosidase assay

β-Galactosidase activity in *S. aureus* was measured using a high-throughput single-step assay in 96-well plates (71). A custom β-galactosidase assay buffer was prepared per 15 mL, containing 60 mM Na_2_HPO_4_, 40 mM NaH_2_PO_4_, 10 mM KCl, 1 mM MgSO_4_, 36 mM β-mercaptoethanol, 1.1 mg/mL ONPG (o-nitrophenyl-β-D-galactopyranoside), 6.7% PopCulture reagent (Millipore #71,092), and 0.4 µL of lysostaphin (10 mg/mL) per reaction. Overnight cultures were diluted 1:10 by transferring 8 µL of culture into 72 µL of 1 × PBS in a fresh 96-well plate. Subsequently, 120 µL of β-galactosidase assay buffer was added to each well. A blank containing 8 µL of TSB plus antibiotic and 72 µL of PBS was included on each plate. Plates were incubated in a microplate reader at 37°C. OD₆₀₀ was measured at the first time point to estimate cell density, and OD₄₂₀ was recorded every 60 s for 1–2 h (20 flashes per well per cycle) with double-orbital shaking at 500 rpm between readings. β-Galactosidase activity was calculated in Miller units using the following formula:


$$\text{Miller units}=\frac{5,000\times \left(\frac{{\text{OD}}_{420}}{\min}\right)}{{\text{OD}}_{600}},$$

where OD_420_/min corresponds to the slope of OD_420_ over time and OD_600_ is the initial reading. Data processing and calculation of Miller units were performed using the software protocol (Gen6).

### Proteomics

Protein samples were first dried using a SpeedVac and subsequently processed using the Micro S-Trap (Protifi) system for tryptic digestion according to the manufacturer’s instructions. Samples were resuspended in SDS lysis buffer (5% SDS, 50 mM TEAB) to solubilize proteins, followed by reduction with TCEP and alkylation with iodoacetamide. After acidification, proteins were bound to S-Trap columns in the presence of methanol-containing binding buffer. Trapped proteins were extensively washed to remove contaminants and detergents before on-column digestion with MS-grade trypsin at a 1:50 enzyme-to-protein ratio. Digestion was carried out overnight at 37°C. Peptides were sequentially eluted using aqueous and organic buffers, pooled, dried, and resuspended in LC-MS-compatible solvent. Samples were clarified by centrifugation, and the supernatant was collected for mass spectrometry analysis. Peptide samples were analyzed by nanoLC-MS/MS using a Thermo Exploris 480 high-resolution mass spectrometer coupled to a Vanquish Neo nanoLC system. One microgram of peptides per sample was loaded onto a trap column and separated on a 50 cm C18 reverse-phase analytical column at a flow rate of 300 nL/min. Peptide separation was achieved using a 90 min linear gradient from 5% to 55% acetonitrile in 0.1% formic acid. Eluting peptides were directly introduced into the mass spectrometer via nanospray ionization. Data were acquired in data-dependent acquisition mode with a 3 s cycle time, high-resolution full MS scans, and subsequent MS/MS fragmentation of selected precursor ions. Singly charged ions, ions with charge states greater than eight, and unassigned charge states were excluded, and dynamic exclusion was enabled to improve proteome coverage. Raw mass spectrometry data were processed using Proteome Discoverer version 3.2 with the Chimerys search engine. Spectra were searched against a *S. aureus* protein database with a precursor mass tolerance of 20 ppm and a fragment mass tolerance of 0.02 Da. Protein quantification was performed using a label-free quantification approach within Proteome Discoverer 3.2. Relative protein abundance was determined by comparing precursor ion intensities across samples, and abundance ratios (fold changes) were calculated as the ratio of mean protein abundance between the different conditions.

### Motif enrichment analysis

The complete *S. aureus* USA300 proteome was obtained from the National Center for Biotechnology Information (https://www.ncbi.nlm.nih.gov/). Target proteins showing differential expression between the wild-type and the *rsaX20*-deficient strains were analyzed for the presence of known metal-binding motifs, including HxH, HxxH, DxH/HxD, and CxxC. Motif scanning was performed using an in-house Python script that identifies motif patterns and annotates potential metal ligands based on conserved residue spacing. For each protein, the number of unique metal-binding motifs and the total motif occurrences were recorded. To assess whether the observed proportion of proteins with metal-binding motifs in the target set was greater than expected by chance, a Monte Carlo resampling approach was applied. Specifically, 100,000 random samples of 88 proteins were drawn without replacement from the full proteome, and the proportion of metal-binding proteins in each sample was calculated to generate a null distribution. The empirical *P*-value was calculated as the fraction of resampled sets with a proportion equal to or greater than the observed proportion. The observed proportion and the resampling distribution were visualized as a histogram with the observed value indicated by a red vertical line. All analyses were performed in R (v4.3.0) using the dplyr, stringr, and ggplot2 packages (72).

## Data Availability

Human chronic wound transcriptome data are available on the Gene Expression Omnibus under identifier GSE319561.

## SUPPLEMENTAL MATERIAL

Data set S1 mbio.00637-26-s0001.xlsx Human samples used in this study.

Data set S2 mbio.00637-26-s0002.xlsx Genomes used to construct the *S. aureus* pangenome.

Data set S3 mbio.00637-26-s0003.xlsx Genes present in the pangenome and their expression in *S. aureus* WT grown *in vitro*.

Data set S4 mbio.00637-26-s0004.xlsx Top 500 genes contributing to variance in human chronic wound/CF sputa compared to BHI and SCFM2.

Data set S5 mbio.00637-26-s0005.xlsx Differential expression analysis of sRNA in human chronic wound and CF sputa compared to *in vitro*.

Data set S6 mbio.00637-26-s0006.xlsx Expression analysis of the nine human-induced sRNAs in 2,177 *S. aureus* transcriptomes.

Data set S7 mbio.00637-26-s0007.xlsx RNAseq analysis of *S. aureus* strains grown in TSB + 40 µM TPEN.

Data set S8 mbio.00637-26-s0008.xlsx Proteomic analysis of *S. aureus* strains grown in TSB + 40 µM TPEN at 6 h.

Data set S9 mbio.00637-26-s0009.xlsx Proteomic analysis of *S. aureus* strains grown in TSB + 40 µM TPEN for 6 or 18 h.

Supporting information mbio.00637-26-s0010.docx Fig. S1 to S7, Table S1, and supplemental references.

ASM does not own the copyrights to Supplemental Material that may be linked to, or accessed through, an article. The authors have granted ASM a non-exclusive, world-wide license to publish the Supplemental Material files. Please contact the corresponding author directly for reuse.
