# Supplementary material for: Discovery of Staphylococcus aureus small RNAs highly expressed during human chronic infection
Source: mBio. 2026 Aug 7;17(9):e00637-26. doi: 10.1128/mbio.00637-26 (PMC13556194; doi:10.1128/mbio.00637-26)
Supplement: Supplementary file 1 [file mbio.00637-26-s0010.docx]

**Supporting Information for**

Discovery of *Staphylococcus aureus* small RNAs highly expressed during human chronic infection.

Paul Briaud, Julia Schumacher and Marvin Whiteley

**Figures**

**Fig. S1: Sample-level transcriptional dispersion within chronic wound, CF sputum, and in vitro sample groups.** Per-gene expression variability is similar across chronic wound, CF sputum, and in vitro conditions. Frequency distribution of standard deviation (rlog-normalized counts) calculated per sRNA gene within each sample group (HCW, n = 5; CF, n = 6; in vitro, n = 10), binned at width 0.1 and expressed as % of sRNAs per bin.

Fig. S2. Principal component analysis *of S. aureus* RNAseq bioprojects. Studies within the black circle were removed from final analysis due to high variance.

Fig. S3. Human-derived *S. aureus* transcriptomes correlate with calprotectin exposed S. aureus transcriptome. Scatter plots comparing log₂ fold-change values for genes differentially expressed in *S. aureus* exposed to calprotectin *in vitro* (Y-axis) (40) against log₂ fold-change values for the same genes in CF sputum (blue) and chronic wound transcriptomes (red, y-axis), relative to *in vitro* growth controls (BHI and SCFM2). Each point represents a single gene shared between the two datasets. Spearman correlation analysis was performed and correlation value (r) indicated. Dot lines represent simple linear regression fits with 95% confidence.

Fig. S4. *rsaX20* sRNA secondary structure. A schematic of the putative *rsaX20* RNA secondary structure was generated using the ViennaRNA tool. Red arrows indicate the start and stop codons of the sORF. The blue box highlights the predicted sRNA seed sequence that potentially binds the Ribosome Binding Sites on mRNAs.

**A**

**B**

Fig. S5: Polar effect of *rsaX20* deletion *(*∆*rsaX20*) on *mdeA* gene. (A) Normalized read count of *mdeA* gene from RNA-seq analysis. (B) RT-qPCR of *mdeA* transcript in the JE2 WT, *rsaX20* deletion (∆*rsaX20*) and the *gfp* insertion strain (∆*rsaX20-gfp*). Expression is normalized to the level of housekeeping gene *hup* transcript. * adjusted *P* < 0.05, ** adjusted *P* <0.01 by one-way ANOVA with Dunnet’s multiple test correction.

Fig. S6: Count of metal-binding motifs in *rsaX20*-regulated proteins. The amino acid sequence of proteins identified by proteomics were examined for conserved metal-binding motifs, including aspartate- and histidine-rich (DxH/HxD/HxH), cysteine-rich (CxxC), and HxxH metalloprotease motifs, as well as ATP-binding signatures indicative of metal-dependent enzymes (FeS cluster). Proteins containing one or more motifs were classified as metal-associated

**A**

**B**

Fig. S7: Translational fusion assays of (A) *paiA* and (B) *adhP* genes. Regions spanning −250 to +99 relative to the start codon (including the native promoter and the first 30 amino acids) were cloned into the pJB185 vector in frame with *lacZ* and transformed into *S. aureus* WT, ∆*rsaX20-gfp*, or sORF* strains. β-galactosidase activity was measured from cells grown in TSB supplemented with 40 µM TPEN. Statistical analysis was performed using a one-way ANOVA corrected with Dunnett’s multiple comparisons test. **** adjusted *P* value < 0.0001.

Table S1: List of strains, plasmids and oligonucleotides used in this study.

| Strain number | description | Source |
| --- | --- | --- |
| PRB104 | *S. aureus* RN4220 | Kreiswirth et al., 1983 |
| PRB9 | *E. coli* IM08B wild type | Monk IR et al., 2021 |
| PRB54 | *S. aureus* JE2 wild type | Fey et al., 2013 |
| PRB75 | PRB54 + pRB6 | This study |
| PRB102 | *S. aureus* JE2 *∆rsaX20* + pPRB93 | This study |
| PRB105 | *S. aureus* JE2 *∆rsaX20* + pPRB6 | This study |
| PRB115 | *S. aureus* JE2 *∆rsaX20-gfp* + pPRB6 | This study |
| PRB116 | *S. aureus* JE2 *∆rsaX20-gfp* + pPRB93 | This study |
| PRB117 | *S. aureus* JE2 sORF* + pPRB6 | This study |
| PRB123 | *S. aureus* JE2 *∆rsaX20-gfp* + pPRB119 | This study |
| PRB134 | *S. aureus* JE2 zur::ery + pPRB85 | This study |
| PRB169 | *S. aureus* JE2 WT + pPRB139 | This study |
| PRB170 | *S. aureus* JE2 *∆rsaX20-gfp* + pPRB139 | This study |
| PRB171 | *S. aureus* JE2 sORF* + pPRB139 | This study |
| PRB181 | *S. aureus* JE2 WT + pPRB150 | This study |
| PRB182 | *S. aureus* JE2 *∆rsaX20-gfp* + pPRB150 | This study |
| PRB183 | *S. aureus* JE2 sORF* + pPRB150 | This study |
| Oligos |  |  |
| OPRB25 | ACAAAAGCTGGGTACC | This study |
| OPRB26 | GGGCGAATTGGAGCTC | This study |
| OPRB11 | GGTACCCAGCTTTTGTTCCCTTTAGTGAGG | García-Nafría J et al., 2016 |
| OPRB12 | GAGCTCCAATTCGCCCTATAGTGAGTCG | García-Nafría J et al., 2016 |
| OPRB116 | cgGGATCCACCCATCAGCATAGTAATAATCA | This study |
| OPRB117 | cgGAATTCGATTAAATATCTTGAAGATGTGTCAGA | This study |
| OPRB85 | GGAATTCCaacacttgtcttgttgtataac | This study |
| OPRB86 | CGGGATCCCGatctctatccctccttgtaaata | This study |
| OPRB157 | GCGGGATCCCAAAAATAAAGCCTTGATAAATTTTA | This study |
| OPRB158 | CGCTCTAGAagtcacctcaaatttaatatca | This study |
| OPRB43 | TATATTTACAAGGAGGGATAGAGAT | This study |
| OPRB44 | TAATACGACTCACTATAGCAAGGAGTGAGTGTATATGC | This study |
| OPRB55 | ATGAACAAAACAGATTTAATCAATGC | This study |
| OPRB56 | TAATACGACTCACTATAGTTATTTTACAGCATCTTTTAATGCTTTAC | This study |
| OPRB190 | acattataaaatagattaaggttaacatttttactg | This study |
| OPRB191 | TAATACGACTCACTATAGtctcaccctttcaaaatttagaaac | This study |
| OPRB187 | aaggatagtgtaagcttaacaataa | This study |
| OPRB188 | TAATACGACTCACTATAGaattacctctcctcatacatg | This study |
| OPRB183 | gacataaatcaatgttctatgctc | This study |
| OPRB184 | TAATACGACTCACTATAGtgactagaatcgaaaaaagctt | This study |
| Plasmids |  |  |
| pPRB6 | pCN51 empty vector | Charpentier et al, 2004 |
| pPRB85 | pJB185_promoter-rsaX20 | This study |
| pPRB93 | pCN51_*rsaX20* | This study |
| pPRB119 | pCN51_*rsaX20*_sORF* | This study |
| pPRB139 | pJB185_*paia* | This study |
| pPRB150 | pJB185_*adhp* | This study |

Dataset S1: List of human samples used in this study (separate file).

Dataset S2: Genomes used to construct the *S. aureus* pangenome (separate file).

Dataset S3: List of genes present in the pangenome and their expression in *S. aureus* WT grown in vitro (separate file).

Dataset S4: Top 500 genes contributing to variance in Human Chronic Wound / CF sputa compared to BHI and SCFM2 (separate file).

Dataset S5: Differential expression analysis of sRNA in Human Chronic Wound and CF sputa compared to in vitro (separate file).

Dataset S6: Expression analysis of the 9 human-induced sRNAs in 2177 *S. aureus* transcriptomes (separate file).

Dataset S7: RNAseq analysis of *S. aureus* WT, ∆*rsaX20* and complement strains grown in TSB + 40µM of TPEN (separate file).

Dataset S8: Proteomic analysis of *S. aureus* WT, ∆*rsaX20* and complement strains grown in TSB + 40µM of TPEN at 6h (separate file).

Dataset S9: Proteomic analysis of *S. aureus* WT, ∆*rsaX20* -*gfp*, complements and sORF* grown in TSB + TPEN 40µM for 6h (cytoplasmic) or 18h (secreted) (separate file).

SI references:

1.	Kreiswirth BN, Lofdahl S, Betley MJ, O'Reilly M, Schlievert PM, Bergdoll MS, et al. The toxic shock syndrome exotoxin structural gene is not detectably transmitted by a prophage. Nature. 1983;305(5936):709-12.

2.	Monk IR, Stinear TPY. From cloning to mutant in 5 days: rapid allelic exchange in Staphylococcus aureus. Access Microbiol. 2021;3(2):000193.

3.	Fey PD, Endres JL, Yajjala VK, Widhelm TJ, Boissy RJ, Bose JL, et al. A genetic resource for rapid and comprehensive phenotype screening of nonessential Staphylococcus aureus genes. mBio. 2013;4(1):e00537-12.

4.	García-Nafría J, Watson JF, Greger IH. IVA cloning: A single-tube universal cloning system exploiting bacterial In Vivo Assembly. Sci Reports. 2016;6(1):27459.

5.	Charpentier E, Anton AI, Barry P, Alfonso B, Fang Y, Novick RP. Novel cassette-based shuttle vector system for gram-positive bacteria. Appl Environ Microbiol. 2004;70(10):6076-85.
